# Plucked!

**Published:** 1890-09-13

**Authors:** 


### The Doctor's Pulpit.

"PLUCKED!"

He can remember the very spot where he received the missive; the colour of the envelope told him his fate. Nevertheless he tore it open and read that, in the dispassionate judgment of the examiners, it was better for him to be referred to his studies for six months. He became conscious that there were thirty or forty other men about him, half of whom had received similar suggestions as to an additional six months' work. He did not offer sympathy to any of them, or seek it from them. The quad was not a pleasant place just then for him, and he thought he had better go home and think, or perhaps smoke. It was the first serious shock that life had met him with.

In itself, it is a small thing to fail to pass a particular examination. To many young men it may be the best thing that can happen. To some, however, it is a disaster as well as a mortification. They have not much money, and do not know how to find the means of living and studying for six months longer. To have passed would have meant wealth; to have failed means shabby clothes, short commons, a grim landlady, poor health, depressed spirits, and a harassing uncertainty as to the result of the next venture. For medical examinations are like those of no other profession. The range of subjects is exceedingly wide, and each subject is a science in itself. The conflict between the examiner and the examined is most unequal. The more intelligent the student, and the wider and deeper his knowledge, the more conscious is he of the inequality. The examiner is a specialist in one subject, and he has been working at that subject almost exclusively for a dozen years at least, perhaps for forty. The examined has to take up ten or twelve different subjects, of most of which he knew nothing whatever before he became a medical student; and yet he has to acquire all the knowledge necessary for passing in every one of them in four or five years. In such circumstances he feels that unless the examiner is thoroughly intelligent, and also sympathetic and impartial, the chance of a pass is more or less of the nature of a "toss-up."

There is no necessary discredit in failure under such circumstances. The lazy man knows, of course, that he has his laziness to thank; but the conscientious worker of average intelligence may justly ascribe his misfortune to bad luck. The cynic, whether he be an examiner or a successful student, does not believe in bad luck; he understands idleness and poor brains, he thinks. But there is no doubt that many a man who fails is scientifically superior, not only to some of those who pass successfully, but even to the examiner himself in his own subject. Not every medical examiner is a philosophic physician; not every lecturer in chemistry or physiology is a true man of science. If examiners as a class were men of broad intelligence as well as of special knowledge, the "crammer" would not find his occupation nearly so profitable. To the plucked man who owes his failure to his idle loafing we have nothing to say except that he richly deserves his fate; and that we hope he will never pass any examining board until he thoroughly mends his ways. It seems to us a great pity that a man should have the chance of passing into the medical profession at all without the approval of those who have learnt to know him by teaching him. Independent examining boards, that is, boards which examine students they have not taught, may be desirable for the purpose of conferring additional qualifications. But no man should be able to enter the medical profession by means of a mere examining board, who has disgraced himself at a medical school.

On the other hand, the conscientious worker, whose star has not been propitious on examination day, should make very light of his trouble. It is nothing! It is a temporary check to his progress, and of no more importance than if his hat were to blow off in the street, and to make him five minutes late in the lecture room. He has made up his mind to get his medical degree and he will get it. The next six months will see such work done that no examiner can pluck him without being unfair. Such a man turns his failure to mental and moral discipline. Like a stream that is checked by a dam on the mountain slope, he gathers force from the obstruction, and either bursts through or overleaps it, on his resistless progress to the great sea. No doubt for the moment he is unhappy, and, perhaps, angry. But that is due to a very large extent to his worried brain, which, from prolonged study, is not in a very fit state to cope with grievous circumstances. If he is a wise man, he will lock up his books, and rest for a month or six weeks. Let him get away from the class-room and from the town, and pay a visit to his mother and sisters, or to his lady-love if he has one. In six weeks he will be ready to enter upon such a course of work as shall make victory certain.

Many are the "plucks" at college, and men forget them. But they never forget the "plucks" and failures of life. It is quite easy to understand why some men fail; others go step by step downwards, of whom it might have been predicted that they would certainly rise. Most men at college have the idea that if once they obtain a medical qualification they will have nothing to do but to march gaily forward to happiness and success. But life does not confirm that idea, except, perhaps, in a few very rare instances. The successful men, in medicine at any rate, have usually been possessed of two qualities—doggedness and patience. They have fought for every inch of ground they have gained, and they have continued to fight in defence of it. It is not really a hardship to have to fight for success, and to wait for it. The hardship is when the summit of success has been too early reached, and there is nothing more to hope for. Medical men have often to wait longer than others for recognition and reward. That is no disadvantage; it is not even a trouble to the well-conditioned. It should be least of all a trouble to the man who has known what it was to be plucked at college, or to have had still more serious reverses in the early part of his professional career. Time is on his side if he have but courage and persistence. Like a noble steeplechaser, whose wind never gives out, he may have stumbled at his first fences and fallen prone into a ditch, whilst his rivals were going ahead almost out of sight. But a good man, like a good horse, cannot be shaken off from the race of life by early reverses, whether small or great. He gathers himself together again; he makes a new start, if need be time after time; and when, at the close of life's examinations, the list of the successful is made up, he is there, and it may be not seldom at the top.

